# iTRAQ-based quantitative proteomic analysis reveals alterations in the metabolism of *Actinidia arguta*

**DOI:** 10.1038/s41598-017-06074-6

**Published:** 2017-07-18

**Authors:** Miaomiao Lin, Jinbao Fang, Xiujuan Qi, Yukuo Li, Jinyong Chen, Leiming Sun, Yunpeng Zhong

**Affiliations:** Zhengzhou Fruit Research Institute, Chinese Academy of Agricultural Sciences, Zhengzhou, He Nan 450009 China

## Abstract

*Actinidia arguta* ‘Tianyuanhong’ is a new kiwifruit variety with an all-red pericarp and pulp, in contrast to the all-green pulp of *A*. *arguta* ‘Yongfengyihao’. Transcriptome profile analysis of fruit color has been reported, however, the metabolic mechanisms producing red flesh remain unknown, and it is unclear why the pulp of ‘Tianyuanhong’ is red rather than green. Herein, we identified differences between the proteomes of two *A*. *arguta* cultivars with different fruit color by using iTRAQ-based quantitative proteomic methods during the stage of color change. In total, 2310 differentially abundant proteins were detected between the two cultivars at 70 and 100 days after flowering, and the protein functions were analyzed based on KEGG and GO. The largest group of differentially expressed proteins were related to photosynthesis, glyoxylate metabolism, N metabolism, and anthocyanin biosynthesis. Finally, to verify the iTRAQ data, 12 representative genes encoding differentially expressed proteins were analyzed via quantitative real-time PCR, and these genes differed in transcriptional and translational expression levels. Our proteomic study contributes to understanding the metabolic pathways and biological processes involved in fruit color changes in different cultivars of *A*. *arguta*. These data and analyses will provide new insight into the development of kiwifruit flesh color.

## Introduction

Kiwifruit (*Actinidia* Lindl.) is valuable in the fruit market because of its unique flavor and abundant nutrients, such as high levels of vitamin C, dietary fiber and amino acids. Kiwifruit includes 54 species and 21 varieties with a wide range of different flesh colors, including green, yellow, red, orange and purple^1–3^. Current commercial kiwifruit varieties are mostly *A*. *chinensis* and *A*. *deliciosa*, as well as *A*. *arguta* and *A*. *eriantha*. Fruit flesh color has become diversified. In recent years, the kiwifruit market has shifted from green fruit flesh to yellow fruit flesh and may now shift to red fruit flesh. Red-fleshed kiwifruit, due to its high level of anthocyanins (ACNs) and antioxidants and health-promoting properties, has interested consumers^4, 5^. The commercial red-fleshed cultivar can be divided into two main types: *A*. *chinensis* cv ‘Hongyang’, which has red pigment only in the inner pericarp and whose fruit requires ripening, and the all-red *A*. *arguta*, which includes the variety ‘Tianyuanhong’, whose fruit is ready to eat.

ACNs, chlorophylls, and carotenoids are the most important pigments responsible for fruit color, including the appearance and attractiveness of the fruit, and they may also provide nutritional value in the form of dietary antioxidants^6–8^. In kiwifruit, the fruit of many species is red. The amounts of individual carotenoids, chlorophylls, and ACNs in red-fleshed *A*. *chinensis* ‘Hongyang’ and *A*. *deliciosa* have been examined, and the pigment of the hybrid offspring of *A*. *arguta* × *A*. *melanandra* has been studied^9–12^; thus, the compounds responsible for pigmentation are known^13–15^. However, while there have been some studies of the mechanism of red fruit color in kiwifruit^16, 17^, little is known about the metabolic pathways producing of red-fleshed kiwifruit.

Proteomics is often used to comprehensively evaluate proteins that respond to a specific treatment; these investigations provide information on both protein quantity and protein activity and identify protein-protein interactions^18^, and proteome changes during fruit ripening and storage have been discussed previously^19^. In recent years, proteomic approaches have been applied to many types of fruit, such as peach, pear, apple, sweet cherry, citrus, and grape^20–25^. Proteomic analysis of kiwifruit was reported previously^26–29^, providing some ideas and insights regarding functions and mechanisms. The complete genome sequence of kiwifruit has helped us to clearly determine gene function^30^. Regarding kiwifruit fruit color, transcriptome analysis has also been reported to explain the metabolic pathway of ACN accumulation; however, the gene and protein expression levels do not correlate well. Therefore, proteomic research is helpful for identifying complex changes in the metabolism of red-fleshed *A*. *arguta*.

In this study, iTRAQ-based proteomic analysis was used to compare quantitative changes in the proteomes of two differently colored varieties of *A*. *arguta* during two fruit development stages. The goals of this study were to compare protein expression patterns and to identify differentially expressed proteins at different stages during the development of *A*. *arguta*. The results of the present study provide a comprehensive understanding of the proteomics of all-red *A*. *arguta* species.

## Results

### ‘Tianyuanhong’ and ‘Yongfengyihao’ fruit development

**Figure 1:**
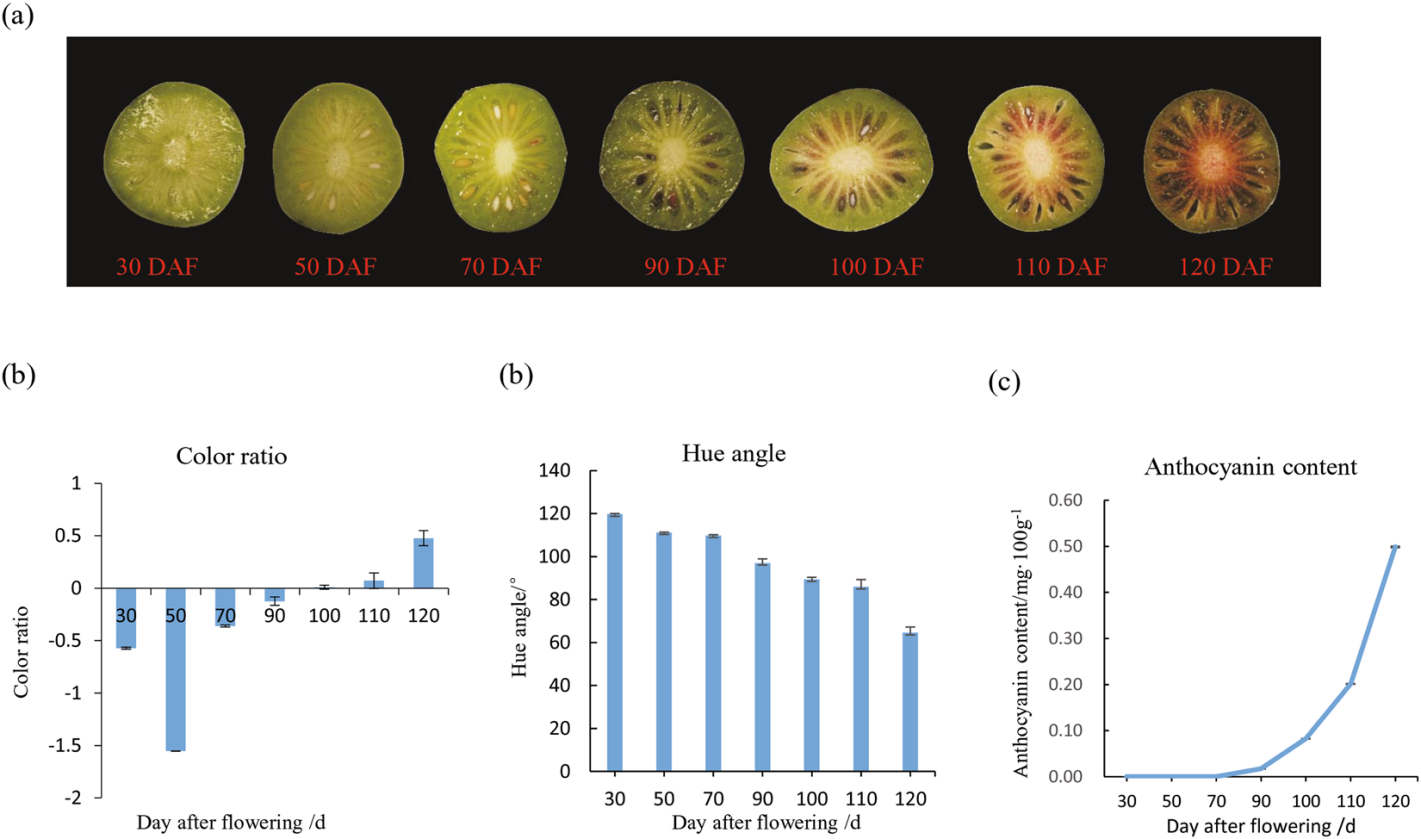
Developmental stages of *A*. *arguta* ‘Tianyuanhong’. (**a**) The changes in fruit color in ‘Tianyuanhong’ during fruit development. (**b**) Changes in the color ratio of ‘Tianyuanhong’ during fruit development. (**c**) Changes in the hue angle of ‘Tianyuanhong’ during fruit development. (**d**) The anthocyanin content in ‘Tianyuanhong’ during fruit development.

‘Tianyuanhong’ fruits were collected at seven stages, ranging from 30 days after flowering (DAF) to 120 DAF (Fig. 1a). The color ratio was negative from 30 to 90 DAF but became positive at 100 DAF, indicating that the fruit color shifted to red (Fig. 1b). The flesh color during early fruit development, from 30 to 90 DAF, showed no obvious changes but began to turn red at 100 DAF. Subsequently, with further fruit development, the fruit color became increasingly red and was the reddest at 120 DAF, as determined based on the h value (Fig. 1c). ACN was first detected at 90 DAF, after which the ACN content gradually increased, peaking at 120 DAF (Fig. 1d). The fruits of ‘Yongfengyihao’ were green throughout fruit development, and no ACN was detected. The pattern of changes in color ratio and hue angle was consistent with that of changes in the ACN content. Our results are similar to those of Seager’s study in that the ACN concentration and hue angle were consistent with the skin and flesh of fruits^12^.

Therefore, we chose 70 DAF, when no ACN was detected, and 100 DAF, when the color ratio became positive, as the stages at which to perform the iTRAQ and qPCR analyses.

### Data analysis and protein identification

In total, 233,500 spectra were identified from the iTRAQ analysis using the two *A*. *arguta* cultivars as the materials. MASCOT generated a total of 13,907 spectra matched to known spectra, 5,535 unique spectra, and 2310 proteins. The distributions of peptide length, peptide count, molecular weight and protein sequence coverage were determined (Supplementary Fig. S1).

### Identification of differentially abundant proteins by iTRAQ

The proteins were screened based on a fold-change value >1.2 and a P-value < 0.05. Based on these criteria, differentially abundant protein species were identified between fruits of the different cultivars and at different stages of fruit development.

The screen comparing 70 and 100 DAF in ‘Tianyuanhong’ identified 229 differentially abundant protein species (DAPS), of which 122 were up-regulated and 107 were down-regulated; these proteins were placed in group B/A. The screen comparing 70 and 100 DAF in ‘Yongfengyihao’ identified 221 DAPS, of which 125 were up-regulated and 96 were down-regulated; these proteins were placed in group D/C.

The comparison between ‘Tianyuanhong’ and ‘Yongfengyihao’ at 70 DAF identified 209 DAPS, of which 100 were up-regulated and 109 were down-regulated; these proteins were placed in group A/C. The comparison between ‘Tianyuanhong’ and ‘Yongfengyihao’ at 100 DAF identified 196 DAPS, of which 94 were up-regulated and 102 were down-regulated; these proteins were placed in group B/D. Detailed information is provided in Supplementary Table 1.

### Bioinformatic analysis of differentially abundant protein species identified by iTRAQ

**Figure 2:**
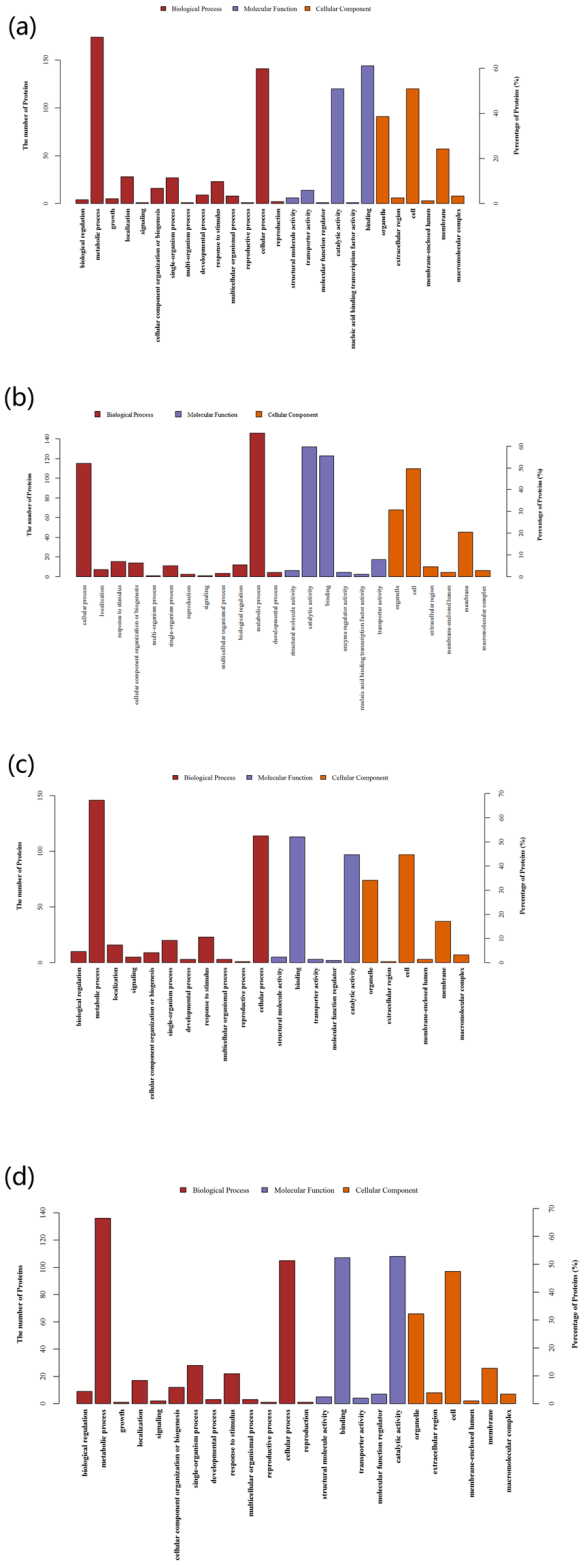
Gene Ontology (GO) annotation of the differentially abundant protein species. (**a**) 70 vs. 100 DAF in ‘Tianyuanhong’ (group B/A). (**b**) 70 vs. 100 DAF in ‘Yongfengyihao’ (group D/C). (**c**) 70 DAF in ‘Tianyuanhong’ vs. ‘Yongfengyihao’ (group A/C). (**d**) 100 DAF in ‘Tianyuanhong’ vs. ‘Yongfengyihao’ (group B/D).

In the comparison between 70 and 100 DAF in ‘Tianyuanhong’ (groups B/A), a total of 229 DAPS were classified into 26 functional groups (Fig. 2a), of which biological processes accounted for 14 GO terms, molecular functions accounted for 6 GO terms, and cellular components accounted for 6 GO terms. The GO categories included ‘binding’ (P = 0.00800), ‘intracellular’ (P = 0.04724), ‘intracellular part’ (P = 0.02790), ‘intracellular organelle’ (P = 0.00960), ‘organelle’ (P = 0.00960), ‘cellular metabolic process’ (P = 0.01518), ‘cytoplasmic part’ (P = 0.01654), ‘intracellular membrane-bounded organelle’ (P = 0.00050), and ‘membrane-bounded organelle’ (P = 0.00050).

In the comparison between 70 and 100 DAF in ‘Yongfengyihao’ (group D/C), a total of 221 DAPS were classified into 24 functional groups (Fig. 2b), of which biological processes accounted for 12 GO terms, molecular functions accounted for 6 GO terms, and cellular components accounted for 6 GO terms. The GO categories included ‘secondary metabolic process’ (P = 6.35E-05), ‘vacuole’ (P = 1.67E-03), ‘sequence-specific DNA binding transcription factor activity’ (P = 9.12E-03), ‘nucleic acid binding transcription factor activity’ (P = 9.12E-03), ‘regulation of gene expression’ (P = 1.29E-02), ‘regulation of metabolic process’ (P = 1.29E-02), ‘regulation of gene expression, epigenetic’ (P = 1.29E-02), and ‘regulation of macromolecule metabolic process’ (P = 1.29E-02).

In the comparison between 70 DAF in ‘Tianyuanhong’ and 70 DAF in ‘Yongfengyihao’ (group A/C), a total of 209 DAPS were classified into 22 functional groups (Fig. 2c), of which biological processes accounted for 11 GO terms, molecular functions accounted for 5 GO terms, and cellular components accounted for 6 GO terms. The GO categories included ‘thylakoid’ (P = 2.09E-12), ‘photosynthesis’ (P = 4.62E-10), ‘plastid’ (P = 0.000323086), ‘intracellular membrane-bounded organelle’ (P = 0.008716782), ‘membrane-bounded organelle’ (P = 0.008716782), ‘nucleoplasm’ (P = 0.022257952), and ‘endosome’ (P = 0.04052163).

In the comparison between 100 DAF in ‘Tianyuanhong’ and 100 DAF in ‘Yongfengyihao’ (group B/D), a total of 196 DAPS were classified into 24 functional groups (Fig. 2d), of which biological processes accounted for 13 GO terms, molecular functions accounted for 5 GO terms, and cellular components accounted for 6 GO terms. The GO categories included (P = 2.65E-05), ‘molecular function regulator’ (P = 0.001777437), ‘enzyme regulator activity’ (P = 0.001777437), ‘thylakoid’ (P = 0.004163896), ‘response to biotic stimulus’ (P = 0.019660177), ‘nucleoplasm’ (P = 0.019689977), ‘secondary metabolic process’ (P = 0.021125983), and ‘DNA binding’ (P = 0.025881387).

In these 4 groups, ‘metabolic and cellular processes’ was the most important term among biological processes, ‘catalytic activity’ and ‘binding’ were the most important terms among molecular functions, and ‘organelle’ and ‘cell’ were the most important terms among cellular components.

**Figure 3:**
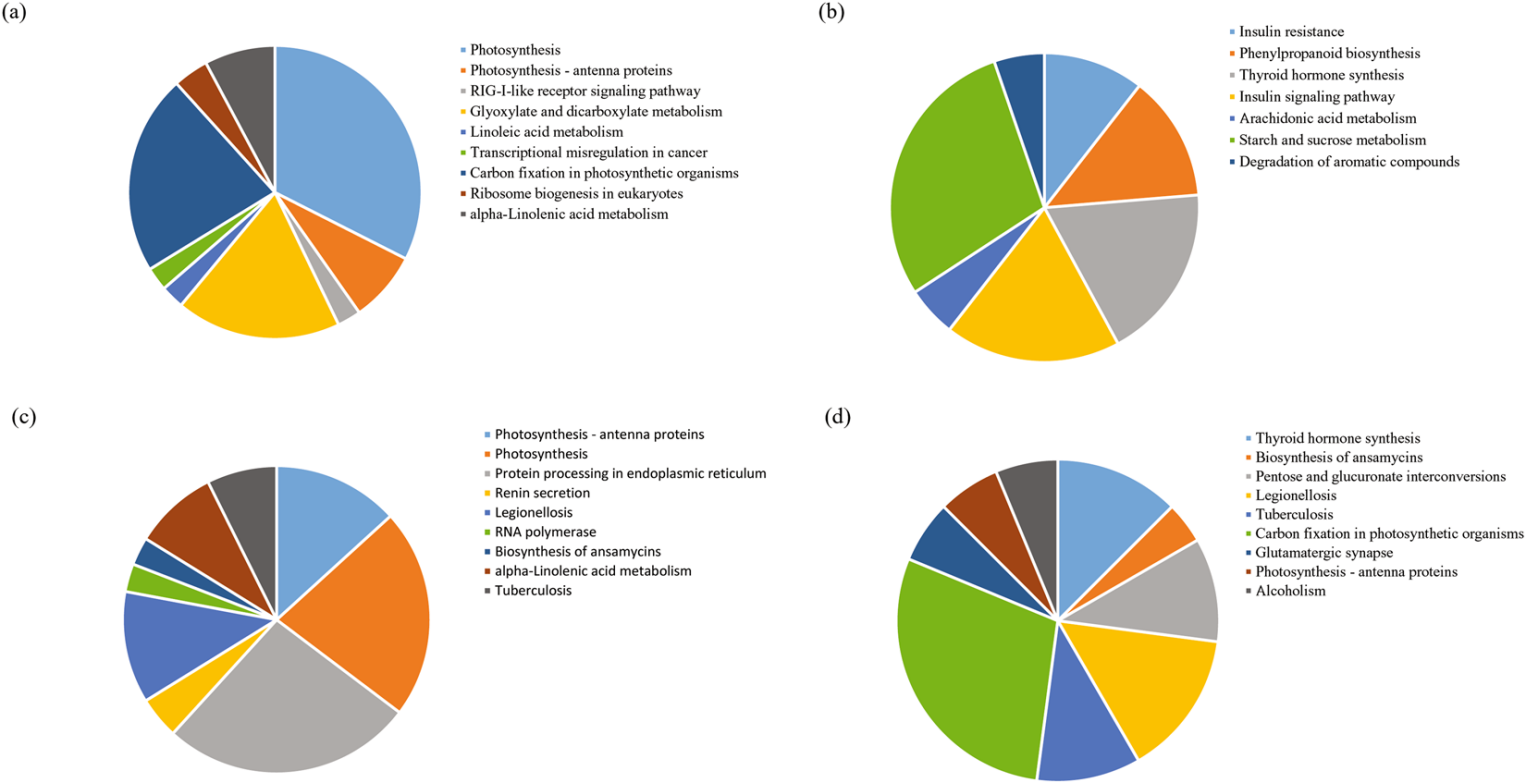
Analysis of KEGG enrichment in the four groups. (**a**) KEGG enrichment in ‘Tianyuanhong’ fruits at 70 vs. 100 DAF (group B/A). (**b**) KEGG enrichment in ‘Tianyuanhong’ vs. ‘Yongfengyihao’ fruits at 70 DAF (group D/C). (**c**) KEGG enrichment in ‘Tianyuanhong’ vs. ‘Yongfengyihao’ fruits at 100 DAF (group A/C). (**d**) KEGG enrichment in ‘Yongfengyihao’ fruits at 70 vs. 100 DAF (group B/D).

To further investigate the biological functions of these proteins, 176 proteins in group B/A were mapped to 155 pathways in the KEGG database. ‘Carbon metabolism’ was the most represented pathway, followed by ‘Photosynthesis’ and ‘Carbon fixation in photosynthetic organisms’. The main pathways in the KEGG enrichment analysis were ‘Photosynthesis’ (P = 1.56E-13), ‘Photosynthesis - antenna proteins’ (P = 0.000248), ‘Carbon fixation in photosynthetic organisms’ (P = 0.016158), ‘Glyoxylate and dicarboxylate metabolism’ (P = 0.024141) and ‘alpha-Linolenic acid metabolism’ (0.045545) (Fig. 3a).

In group D/C, 133 proteins were mapped to 148 pathways in the KEGG database. ‘Carbon metabolism’ was the most represented pathway, followed by ‘Starch and sucrose metabolism’ and ‘Biosynthesis of amino acids’. The main pathways in the KEGG enrichment analysis were ‘Insulin resistance’ (P = 0.00628), ‘Phenylpropanoid biosynthesis’ (P = 0.043731), ‘Thyroid hormone synthesis’ (P = 0.008421), ‘Insulin signaling pathway’ (P = 0.003445) and ‘Starch and sucrose metabolism’ (P = 0.017741) (Fig. 3b).

In group A/C, 136 proteins were mapped to 143 pathways in the KEGG database. ‘Carbon metabolism’ was the most represented pathway, followed by ‘Protein processing in endoplasmic reticulum’ and ‘Photosynthesis’. The main pathways in the KEGG enrichment analysis were ‘Photosynthesis’ (P = 1.07E-05), ‘Photosynthesis - antenna proteins’ (P = 1.59E-08), ‘Protein processing in endoplasmic reticulum’ (P = 0.005526), ‘alpha-Linolenic acid metabolism’ (P = 0.032945) and ‘RNA polymerase’ (P = 0.022258) (Fig. 3c).

In group B/D, 131 proteins were mapped to 171 pathways in the KEGG database. ‘Carbon metabolism’ was the most represented pathway, followed by ‘Carbon fixation in photosynthetic organisms’ and ‘Biosynthesis of amino acids’. The main pathways in the KEGG enrichment analysis were ‘Pentose and glucuronate interconversions’ (P = 0.021292), ‘Carbon fixation in photosynthetic organisms’ (P = 0.047208), ‘Photosynthesis - antenna proteins’ (P = 0.049349), ‘Thyroid hormone synthesis’ (P = 0.016747), and ‘Glutamatergic synapse’ (P = 0.049349) (Fig. 3d).

### Comparison of proteins with differential expression in ‘Tianyuanhong’ and ‘Yongfengyihao’

**Figure 4:**
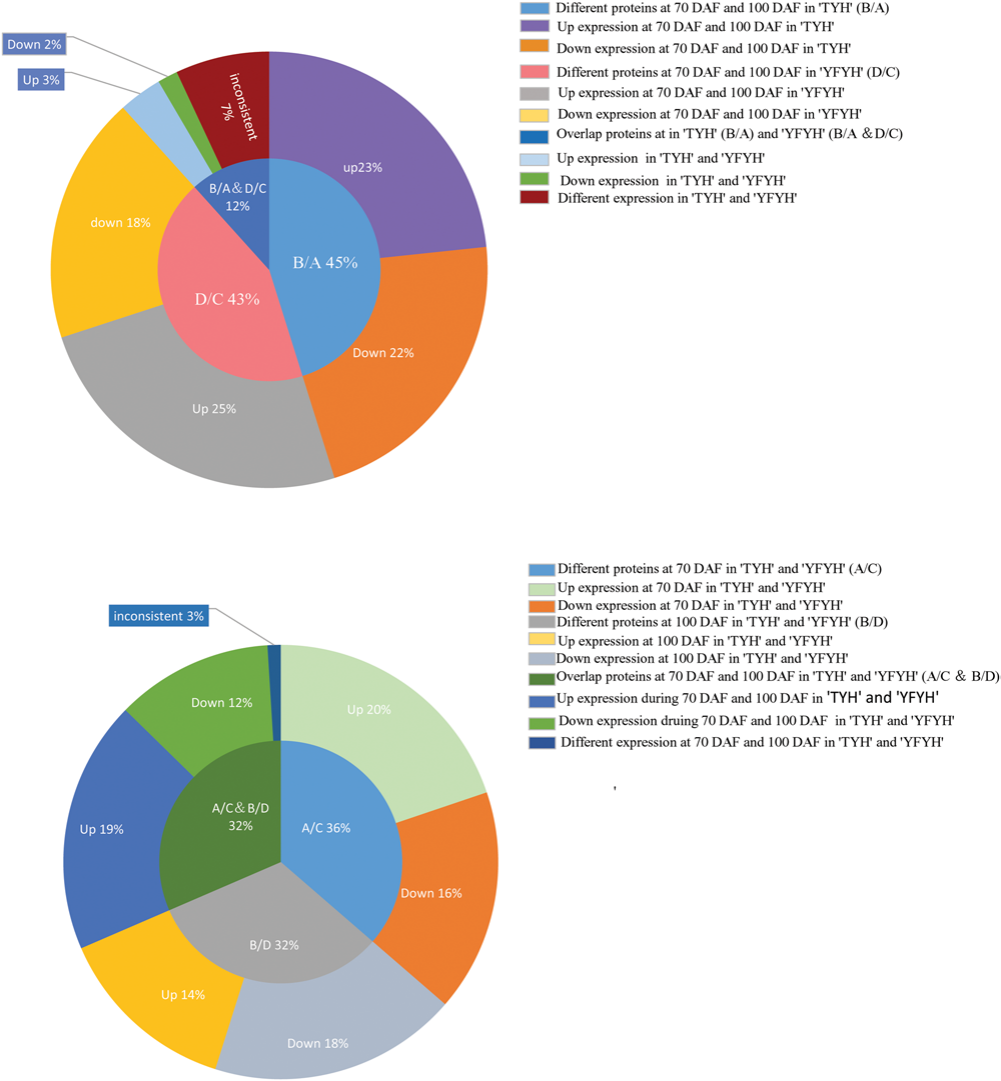
Venn diagram of the differentially abundant protein species in the 4 groups. (**a**) Comparison of proteins in ‘Tianyuanhong’ and ‘Yongfengyihao’ at 70 days and 100 days. (**b**) Comparison of proteins at 70 DAF in ‘Tianyuanhong’ and ‘Yongfengyihao’ and 100 DAF in ‘Tianyuanhong’ and ‘Yongfengyihao’.

To understand the mechanism for producing red flesh, proteins showing differential expression at the color-change stages in *A*. *arguta* with red flesh (group B/A) and with green flesh (group D/C) were evaluated. In all, there were 182 specific proteins in group B/A (45%) and 174 (43%) specific proteins in group D/C (Fig. 4a); only 47 proteins (12%) showed differential expression in both the two cultivars at the same time.

Comparing group A/C with group B/D, representing the states before and after the color change stage, respectively, there was an overlap of a total of 97 (32%) DAPS, of which 58 (19%) were up-regulated and 36 (12%) were down-regulated; 3 (3%) DAPS showed the opposite trend in the two cultivars. There were 112 (36%) differentially expressed proteins in group A/C, of which 61 (20%) were up-regulated and 51 (16%) were down-regulated. A total of 99 DAPS (32%) were observed in group B/D, of which 57 (18%) were up-regulated and 42 (14%) were down-regulated (Fig. 4b).

### Transcriptional expression analysis by qRT-PCR

**Figure 5:**
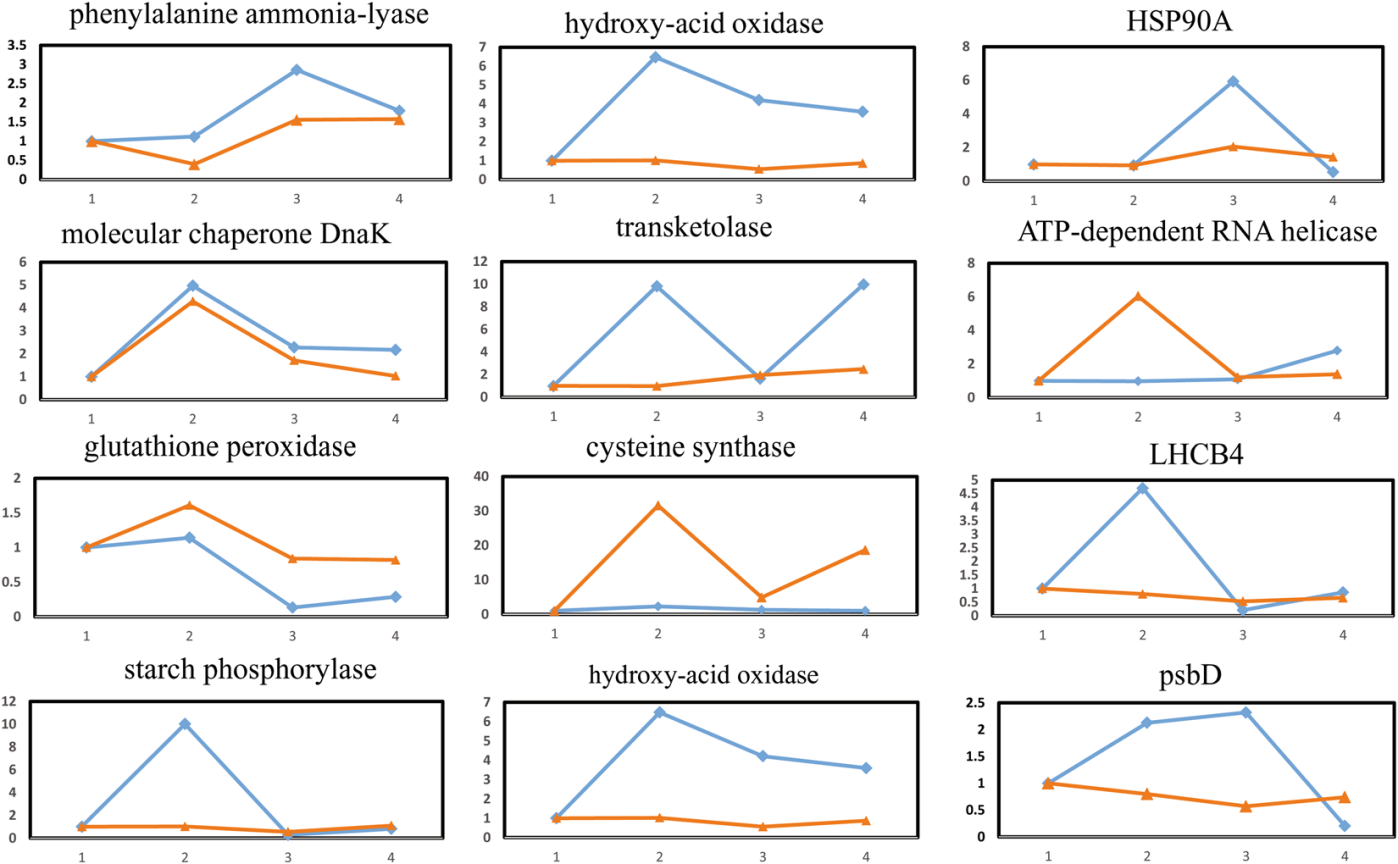
Comparative analysis of the transcript and protein levels of the differentially abundant proteins at two stages of fruit development in ‘Tianyuanhong’ and ‘Yongfengyihao’.

To evaluate the agreement between mRNA transcript level and protein abundance, 12 genes encoding proteins identified in the previous analyses were used for quantitative real-time polymerase chain reaction (qRT-PCR) verification of the dynamic transcriptional expression levels. Figure 5 shows that the expression levels of 8 genes, including phenylalanine ammonia-lyase, molecular chaperone DnaK, and hydroxypyruvate reductase, were similar to the abundances of the corresponding proteins. In contrast, in ‘Yongfengyihao’, the expression levels of LHCB4 and starch phosphorylase showed the opposite trends as the abundances of their corresponding proteins; two genes, those encoding hydroxyl-acid oxidase and psbD, showed the opposite trends in both cultivars. The differences between the two cultivars and the inconsistency between the expression levels of the four genes and the protein abundances probably resulted from various posttranslational modifications.

## Discussion

In this study, we performed iTRAQ analysis on the flesh of kiwifruit from two *A*. *arguta* cultivars at two stages of fruit development. Proteomics analysis provides a broad perspective on the process of fruit color changes, which prompts us not only to pay attention to the ACN biosynthesis pathway but also to further evaluate the upstream changes related to the red color of flesh in *A*. *arguta*. The results provide detailed proteomic information about the development of green flesh and red fruit flesh in *A*. *arguta*; 2310 proteins were identified during the fruit color changes stages at these two cultivars. During the color-change stages, the green- and red-fleshed *A*. *arguta* show differences in the most abundant proteins, with photosynthesis and energy metabolism, C metabolism, N metabolism, and ACN biosynthesis showing high expression in red-fleshed *A*. *arguta*. This analysis helped us to clearly identify proteins associated with fruit flesh color in *A*. *arguta* and to determine the mechanism by which red fruit color forms for use in breeding.

### Proteins involved in energy metabolism and carbon metabolism

Light is the key stimulus for ACN biosynthesis^31–33^; the positive and negative regulation of ACN pigmentation by light has important physiological implications^34^. Photosynthesis is the decisive factor in sugar synthesis and transport and connects the environmental and biological factors that regulate fruit development, including the biosynthesis of ACN^35^. Photosynthesis generated the raw material triosephosphate for amino acid biosynthesis during the stage of color changes (70 DAF to 100 DAF, group B/A) in ‘Tianyuanhong’. Several proteins involved in photosynthesis were up-regulated: photosystem II PsbH protein; photosystem I subunits PsaN, II, III, IV, VI, and XI; F-type H+ -transporting ATPase subunit beta; oxygen-evolving enhancer proteins 1, 2, and 3; and rubisco. Light-harvesting complex II chlorophyll a/b binding proteins 1-6 were also up-regulated. The up-regulated proteins also reflect the fact that chlorophyll levels decrease during skin ripening. However, in ‘Yongfengyihao’ at the color-change stages, there were no obvious changes in photosynthesis proteins. As ‘Yongfengyihao’ has green flesh and ‘Tianyuanhong’ has red flesh, the formation of the red color may be related to photosynthesis during the color-change stages. Comparing ‘Tianyuanhong’ with ‘Yongfengyihao’ at 100 DAF (group B/D), rubisco was up-regulated; at 70 DAF (groups A/C), light-harvesting complex II chlorophyll a/b binding protein was down-regulated, along with some photosystem II proteins. Acetyl-CoA C-acetyltransferase was up-regulated in ‘Tianyuanhong’.

**Figure 6:**
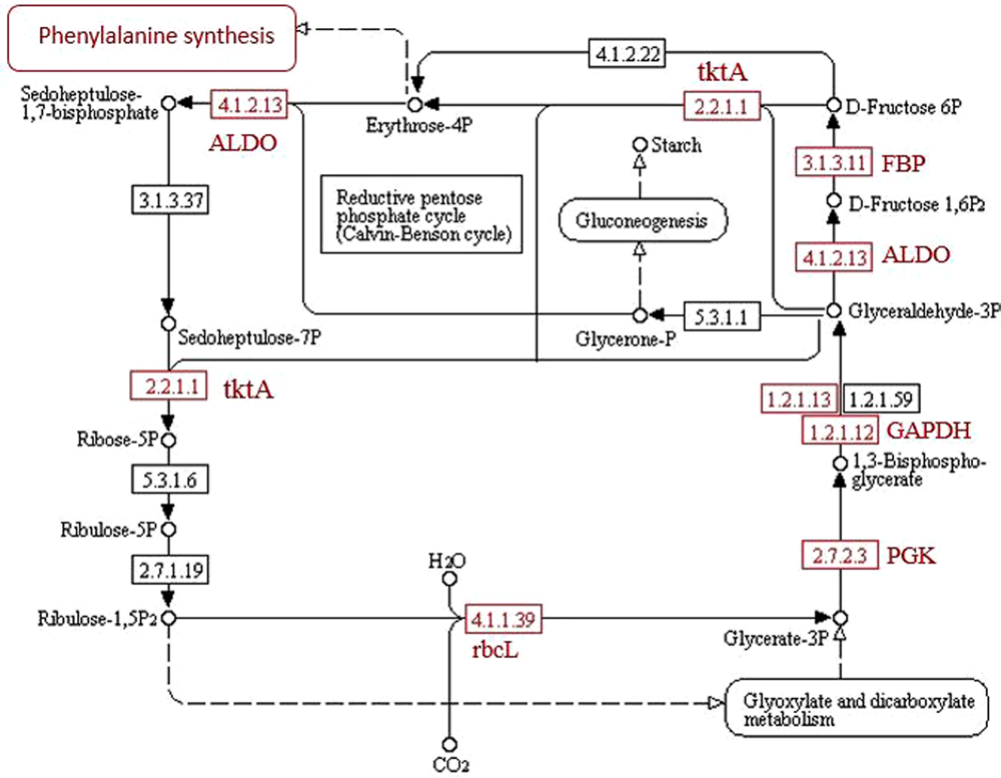
Main protein changes relating to carbon fixation in photosynthetic organisms.

In these two varieties, one of the pathways showing the most marked differences is carbon fixation in photosynthetic organisms, through which carbon dioxide is fixed, and after undergoing a series of synthetic reactions, is incorporated into the phenylalanine pathway. In association with this process, the proteins ribulose-bisphosphate aldolase (rbcL), phosphoglycerate kinase (PGK), transketolase (tktA), fructose-1,6-bisphosphatase (FBP), and fructose-bisphosphate aldolase (ALDO) were all up-regulated in ‘Tianyuanhong’ at 100 DAF in contrast to 70 DAF (group B/A). However, we could not detect changes in these proteins in ‘Yongfengyihao’ between these two stages (Fig. 6).

Sugars play a major role in ACN biosynthesis in numerous plant species^36–38^. Ohto reported that *Arabidopsis* cultured on Suc-containing medium produced a high amount of ACNs^39^; sugar content may play an essential role in ACN accumulation. Two glycosyltransferases were up-regulated at 100 DAF in ‘Tianyuanhong’ (group B/A); much of the diversity of ACNs is due to the action of glycosyltransferases, which add sugar moieties to ACNs. The great diversity of ACN compounds comes from the glycosylation of the basic flavonol structure^40, 41^. At 100 DAF in ‘Yongfengyihao’ (group D/C), glycosyltransferase showed no obvious changes. Comparing ‘Tianyuanhong’ with ‘Yongfengyihao’ at 100 DAF (group B/D), glycosyltransferase was up-regulated. ‘Starch and sucrose metabolism’ proteins were also down-regulated, especially starch phosphorylase, 1,4-alpha-glucan branching enzyme, 4-alpha-glucanotransferase and sucrose synthase.

Citric acid plays a key role in fruits and vegetables; it helps to maintain apple fruit development, and its application maintains the red color of apple fruits^42^. Citrate synthase was up-regulated at 100 DAF in ‘Tianyuanhong’ (group B/A), but there were no obvious protein changes in ‘Yongfengyihao’.

At 70 DAF and 100 DAF in ‘Tianyuanhong’, hydroxyl-acid oxidase (HAO) was up-regulated; HAO can catalyze the conversion of oxygen to hydrogen peroxide, which is known to be a raw material for glutathione metabolism and is involved in glutamine synthesis through the pentose phosphate pathway. When comparing samples at 100 DAF in ‘Tianyuanhong’ and ‘Yongfengyihao’, transketolase (tkt A) was present at a higher level in ‘Tianyuanhong’, accelerating glutamine synthesis.

### Proteins involved in amino acid metabolism

Amino acids are the main raw material for the ACN biosynthesis, and amino acid metabolism influences phenylpropanoid metabolism, which activates ACN biosynthesis^43^. Glutamine synthetase was up-regulated at 100 DAF to nearly 3 times the level at 70 DAF in ‘Tianyuanhong’ (group B/A). Glutamate is an important intermediate in nitrogen metabolism, and glutamine synthetase can catalyze the conversion of glutamate into glutamine. A similar result has been reported in grape, in which the glutamate content was constant from flowering to color change and then diminished^44^. During fruit ripening stages, glutamine synthetase in fruit skin cells catalyzes the conversion of a large amount of glutamate due to the increased nitrogen requirement^25^.

ACNs are accumulated via the phenylpropanoid metabolism pathway, which is initiated by the conversion of phenylalanine into cinnamic acid by phenylalanine ammonia lyase (PAL) and then diverges into various branches at ρ-coumaroyl CoA, leading to the synthesis of flavonols, cyanidins, and ACNs^43^. In our study, three PAL proteins were identified. PAL proteins in ‘Yongfengyihao’ were up-regulated at 70 DAF and down-regulated at 100 DAF. This suggests a block of the ACN biosynthesis. In grape, the *PAL1* gene expression level was higher during the later maturation phases, and the enzymatic activity of PAL was approximately two-fold higher in mature or fully mature berries, suggesting regulation at the transcriptional level^45^.

### Proteins involved in regulating gene expression

Heat shock proteins (HSPs) stabilize both old and new proteins. We identified HSPA1_8, HSP20, HSP90A, and HSP90B as up-regulated at 70 DAF in ‘Tianyuanhong’ compared with ‘Yongfengyihao’ (group A/C). This evidence is consistent with Nakayama’s results regarding flower color in which HSPs showed high expression levels^46^.

**Figure 7:**
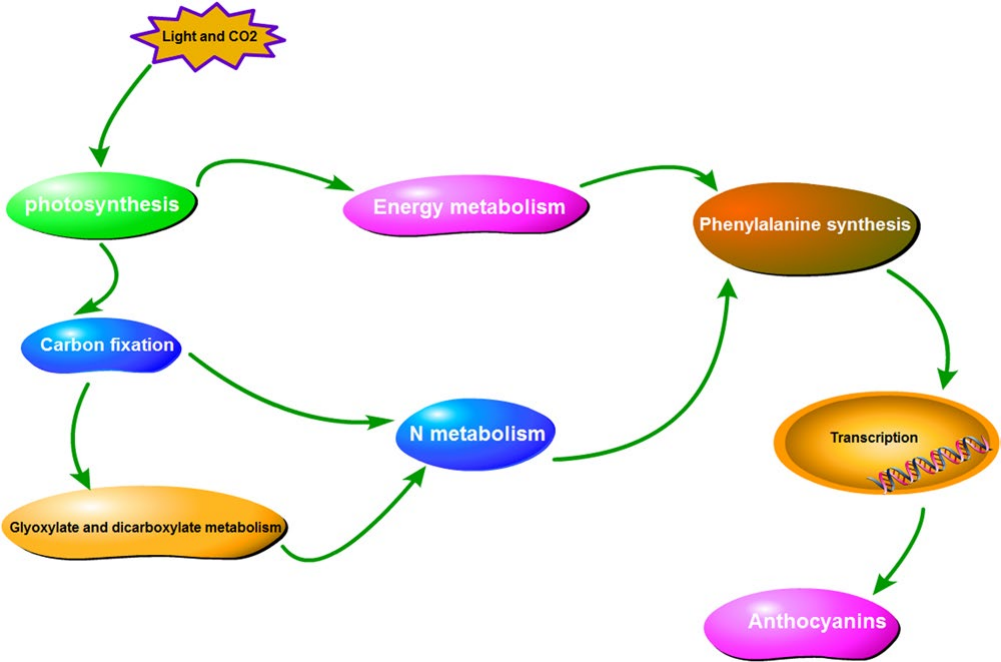
The key metabolic pathway in ‘Tianyuanhong’ during the stage of fruit color change.

In conclusion, we investigated the changes in protein expression levels during fruit flesh color changes at 70 and 100 DAF in two *A*. *arguta* cultivars. The results of this research suggest that photosynthesis and carbon fixation were the main causes of color changes that resulted in the green and red flesh in *A*. *arguta*. All the differentially abundant proteins suggested an association with ACN biosynthesis, a simple pathway that is shown in Fig. 7, and the detailed expression levels of the proteins in this figure are shown in supplementary Table 2. We detected differentially expressed proteins only upstream of the ACN biosynthetic pathway, which indicates that color changes will involve changes in ACN biosynthesis, and the changes in protein abundance may appear somewhat later than the changes in fruit color. The results of the present study provide novel clues that facilitate a better understanding of the metabolic network of *A*. *arguta* fruit color development.

## Materials and Methods

### Plant materials

*A*. *arguta* ‘Tianyuanhong’, with all-red fruit and pericarp, was selected by Zheng Zhou Fruit Research Institute, and *A*. *arguta* ‘Yongfengyihao’, with all-green fruit and pericarp, was cultured in Liaoning Province, Da Lian City. Fruits were collected at 30, 50, 70, 90, 100, 110 and 120 DAF, cut into small pieces, quickly frozen in liquid nitrogen, and stored at −80 °C for ACN measurement and iTRAQ-based and q-PCR analyses.

### Measurement of a and b values

The value a represents red and green indicators, while the value b represents yellow and blue indicators. These values were used to calculate h (color ratio) using the formula h = a/b and h* (hue angle) using the formula h* = arc [tan (b/a)] and were measured in fruit samples at 8 stages of fruit development using a portable color meter (CR-400, Japanese Konica Minolta).

### Anthocyanin extraction and evaluation by HPLC

A-solution (formic acid/methanol (1:49 v/v)) and B-solution (formic acid/methanol/H_2_O (1:5:44 v/v/v)) were used for ACN extraction. Fruit samples at 7 stages of fruit development (2 g fresh weight) were ground to powder in liquid nitrogen, mixed with 6:1 (v/w) A-solution, oscillated for 10 min, ultrasonicated (40 °C, 40 kHz, 100 w) for 10 min, and centrifuged (4 °C, 8000 r·min^−1^) for 10 min. The residues were re-extracted three times. Then, 10 ml of the collected supernatant was dried in a Rotary Evaporator (RE-52 AA, Shanghai Yarong Biochemistry Instrument Factory), dissolved in 2 ml of B-solution, filtered through a 0.22 μm filter membrane and retained for HPLC analysis^47^.

HPLC was performed to detect ACNs. The parameters were as follows: reversed phase column 4.6 × 250 mm, 5 m (Agilent C18); column temperature 25 °C; injection volume 10 ml; flow rate 1.0 ml/min; and detection wave length 525 nm. The linear gradient was (proportion of A-solvent) 0–4 min, 6–10%; 4–12 min, 10–25%; 12–13 min, 25–40%; 13–15 min, 40–100%; 15–20 min, 100–6%; and 20–30 min, 6–6%. Cyanidin-3-galactoside (Solarbio, Beijing) was used as the authentic standard to generate the standard curve^48^.

### Protein extraction

Equal amounts of fruit sample were ground into fine powder in liquid nitrogen, Then, a 5-fold volume of TCA/acetone (1:9) was added to the powder and mixed by vortexing. The mixture was placed at −20 °C for more than 4 h precipitation. Next, the sample was centrifuged at 6000 g for 40 min at 4 °C, and the supernatant was discarded. Pre-cooled acetone was added and washed three times. The precipitant was air dried. Then, a 30-fold volume of SDT buffer was added to 20–30 mg of powder, mixed and boiled for 5 min. The lysate was sonicated and then boiled for 15 min. After centrifugation at 14,000 g for 40 min, the supernatant was filtered using a 0.22 μm filter, and the filtrate was collected. Protein quantification was performed using the BCA Protein Assay Kit (Bio-Rad, USA). The samples were then assembled and stored at −80 °C.

### Filter-aided sample preparation (FASP digestion)

For each sample, 30 μL of protein solution was added into DTT (1,4-dithio-dl-threitol), with a final concentration of 100 mM, followed by incubation in a boiling water bath for 5 min and then cooling to room temperature. Then, 200 μL of UA buffer (8 M urea, 150 mM Tris-HCl pH 8.0) was added, followed by mixing into a 10 kD ultrafiltration centrifuge tube and centrifugation at 14,000 g for 15 min, after which the filtrate was discarded (and this step was repeated once). Next, 100 μL of IAA buffer (100 mM IAA in UA) was added, shaken at 600 rpm for 1 min, protected from light at room temperature for 30 min, and centrifuged at 14,000 g for 15 min. The entire procedure described above were repeated twice with the addition of 100 μL UA buffer by centrifugation at 14,000 g for 15 min. This procedure was repeated twice by adding 100 μL of a 10-fold diluted dissolution buffer and centrifuging 14,000 g for 15 min. Then, 40 μL of Trypsin buffer (4 μg Trypsin in 40 μL of dissolution buffer) was added, followed by shaking at 600 rpm for 1 min and incubation at 37 °C for 16–18 h. Next, the samples were centrifuged at 14,000 g for 15 min, followed by the addition of 40 μL of 10-fold-diluted dissolution buffer, centrifugation at 14,000 g for 15 min, and collection of the filtrate. Peptide fragments were desalted using a C_18_ cartridge. Peptide fragments were lyophilized and reconstituted with 40 μL of dissolution buffer. Then, the peptide was quantitated (OD280)^49^.

### iTRAQ labeling and SCX fractionation

Each sample included 100 μg of peptide, according to the instructions for the AB SCIEX iTRAQ labeling kit for labeling. Each set of labeled peptides was mixed and graded using AKTA Purifier 100 (GE Healthcare). Buffer A solution was 10 mM KH_2_PO_4_, 25% ACN, pH 3.0, and buffer B solution was 10 mM KH_2_PO_4_, 500 mM KCl, 25% ACN, pH 3.0. The column was equilibrated with liquid A (10 mM KH_2_PO4, 25% ACN, pH 3.0, B solution 10 mM KH_2_PO_4_, 500 mM KCl, 25% ACN, pH 3.0), and the sample was transferred from the injector to the column (5 μm, 200 Å, PolyLC Inc., Maryland, U.S.A.) for separation at a flow rate of 1 ml/min from 0 min–22 min. Liquid B (10 mM KH_2_PO_4_, 500 mM KCl, 25% ACN, pH 3.0) was increased at a linear gradient from 0% to 8% from 22 min–47 min, at a linear gradient from 8% to 52% from 47 min–50 min and at a linear gradient from 52% to 100% from 50 min–58 min, after which liquid B was maintained at 100%; 58 min later, solution B was reset to 0%. The absorbance value at 214 nm was monitored during the elution, the eluted fraction was collected every 1 min, and approximately 30 samples of the eluted fraction were collected. Samples were combined into 15 groups, respectively, after being freeze-dried with C18 Cartridge (Empore^TM^ SPE Cartridges C18 (standard density) for desalination.

### LC-MS/MS analysis

The samples were analyzed using a Q Exactive mass spectrometer (Thermo Scientific). The analysis time was 60 min, the detection mode was positive ion, the range of the parent ion was 300–1800 m/z, the resolution of the first mass spectrometer was 70,000 at 200 m/z, the automatic gain control (AGC) target was set to 3 e6, the maximum injection time was set to 10 ms, the number of scan ranges was 1, and the dynamic exclusion was 40.0 s. The mass-to-charge ratios of peptides and polypeptide fragments were collected as follows: 10 fragment maps were acquired after a full scan (MS2 scan), the MS2 Activation Type was HCD, the isolation window was 2 m/z, 17,500 at 200 m/z, the number of microscans was 1, the maximum injection time was 60 ms, the normalized collision energy was 30 eV, and the underfill was 0.1%.

### Protein identification and quantification

MS/MS spectra were searched using MASCOT (Matrix Science, London, UK; version 2.2) embedded in Proteome Discoverer 1.4 and run against the Ericales protein database (released 2015-10-15 and including 21,318 sequences). The research parameters were as follows: Trypsin/P was specified as the enzyme, and two missed cleavages were permitted. The fixed modification was carbamidomethyl (C), the variable modifications were oxidation (M) and iTRAQ 4/8-plex (Y), the peptide mass tolerance was ±20 ppm, and the fragment mass tolerance was 0.1 Da. The results were then filtered according to a false discovery rate (FDR) of ≤0.01. The protein ratios were calculated as the median of only unique peptides of the protein. Normalizing all peptide ratios based on the median protein ratio, the median protein ratio should be 1 after the normalization.

### Bioinformatics functional analysis

The sequence data of the selected differentially expressed proteins were retrieved in batches from UniProtKB database (Release 2015_10) in FASTA format. The retrieved sequences were locally searched against the SwissProt database (mammals) using the NCBI BLAST+ client software (ncbi-blast-2.2.28+-win32.exe) to identify homologous sequences from which the functional annotation could be transferred to the studied sequences. In this work, the top 10 blast hits with E-value < 1e-3 for each query sequence were retrieved and loaded into Blast2GO^50^ (Version 2.7.2) for GO^51^ mapping and annotation. Following annotation and annotation augmentation steps, the studied proteins were blasted against KEGG GENES to retrieve their KOs and were subsequently mapped to pathways in KEGG^52^.

### Verification of iTRAQ data by qPCR

Total RNA was extracted from ‘Tianyuanhong’ and ‘Yongfengyihao’ fruits at 70 and 100 DAF using CTAB methods. A total of 2 µg RNA was reverse transcribed to cDNA using a cDNA synthesis kit (Fermentas Canada). qPCR was performed using the LightCycler® 480 Real-Time PCR system. The target gene primers were designed using Primer Express 5.0, and the actin gene was selected as the reference gene. Every primer was used 3 times. The relative expression levels were calculated using 2^−ΔΔCt^ method^53^, and the primers are shown in Supplementary Table 3.

## Electronic supplementary material

Supplementary information

Figure S1

Table S1

Table S2

Table S3
