# Supplementary material for: iTRAQ-based quantitative proteomic analysis reveals alterations in the metabolism of Actinidia arguta
Source: Sci Rep. 2017 Jul 18;7:5670. doi: 10.1038/s41598-017-06074-6 (PMC5515984; doi:10.1038/s41598-017-06074-6)
Supplement: Supplementary file 1 — Supplementary information [file 41598_2017_6074_MOESM1_ESM.doc]

**Supporting Information for**

**iTRAQ-based quantitative proteomic analysis reveals alterations in the metabolism of *Actinidia arguta***

**Authors:** Miaomiao Lin, Jinbao Fang^*^, Xiujuan Qi^*^ , Yukuo Li, Jinyong Chen, Leiming Sun, Yunpeng Zhong

**Affiliation:** Zhengzhou Fruit Research Institute, Chinese Academy of Agricultural Sciences, Zhengzhou, Henan, 450009, China

*Corresponding author: Jinbao Fang, Xiujuan Qi, Chinese Academy of Agricultural Sciences, Henan, 450009, China, Tel: 0371-65330995, E-mail: [fangjinbao@caas.cn](mailto:fangjinbao@caas.cn), [qixiujuan@caas.cn](mailto:qixiujuan@caas.cn)

Figure S1. The distributions of peptide length (a), peptide count (b), molecular weight (c), and protein sequence coverage (d) were determined by iTRAQ analysis.

Table S1. Proteins quantitative at 70 DAF and 100 DAF in ‘Tianyuanhong’ vs. ‘Yongfengyihao’

Table S2. Proteins showing KEGG enrichment at 70 DAF and 100 DAF in ‘Tianyuanhong’ vs. ‘Yongfengyihao’

Table S3. The primers in q-PCR
