# Supplementary material for: iTRAQ-based quantitative proteomic analysis reveals alterations in the metabolism of Actinidia arguta
Source: Sci Rep. 2017 Jul 18;7:5670. doi: 10.1038/s41598-017-06074-6 (PMC5515984; doi:10.1038/s41598-017-06074-6)
Supplement: Supplementary file 2 — Figure S1 [file 41598_2017_6074_MOESM2_ESM.doc]

**iTRAQ-based quantitative proteomic analysis reveals alterations in the metabolism of *Actinidia arguta***

**Authors:** Miaomiao Lin, Jinbao Fang^*^, Xiujuan Qi^*^ , Yukuo Li, Jinyong Chen, Leiming Sun, Yunpeng Zhong

　 (a) (b)


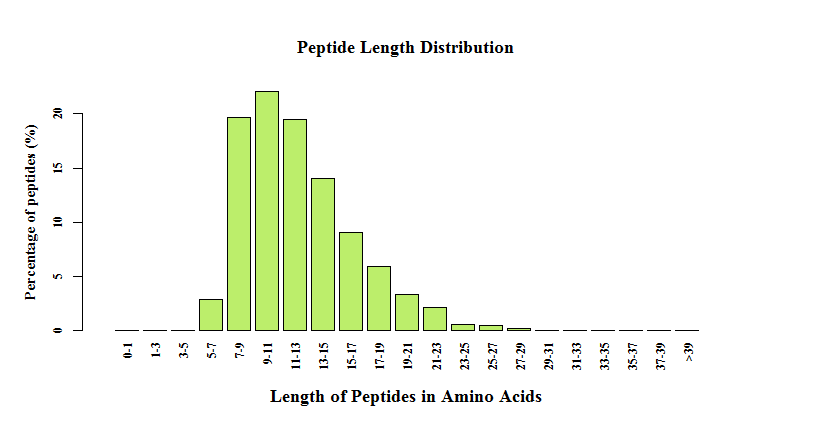

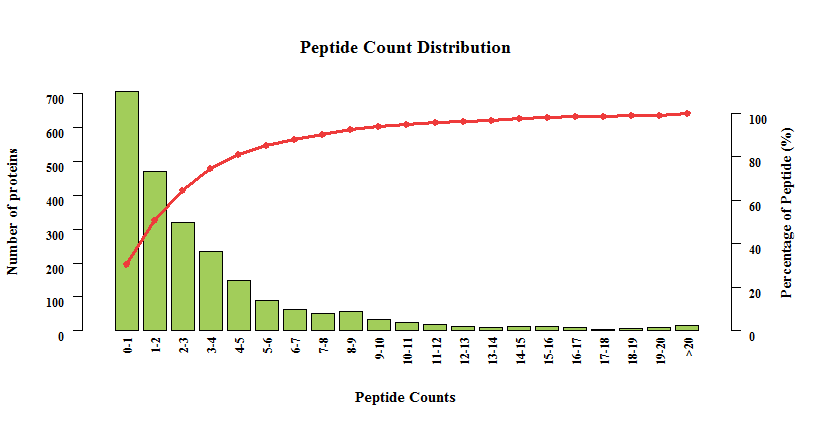


　(c) (d)


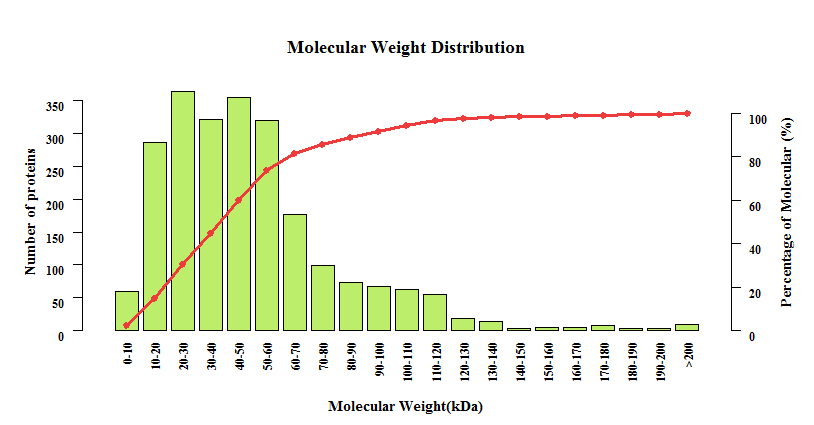

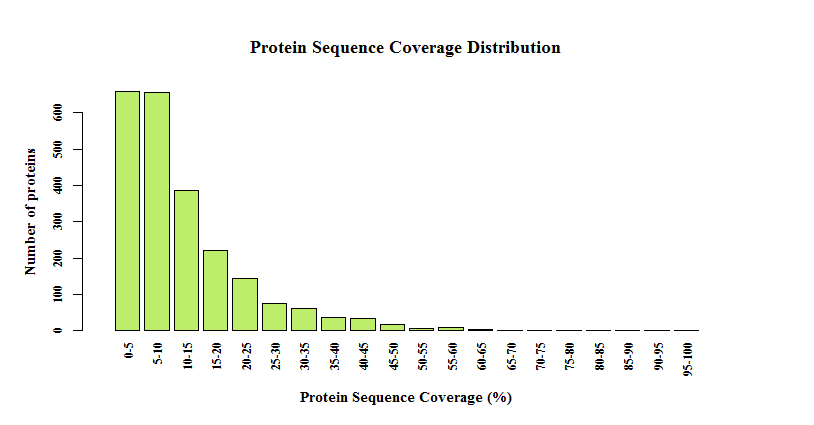


Fig. S1. The distributions of peptide length (a), peptide count (b), molecular weight (c), and protein sequence coverage (d) were determined by iTRAQ analysis.
